# Correlations between endocrine-metabolic characteristics and body fat distribution, appetite, growth, and memory in children with Prader-Willi syndrome

**DOI:** 10.1016/j.clinsp.2026.101042

**Published:** 2026-07-02

**Authors:** Ying Zhang, Xiang Li, Jian Wen, Yong Rao, Juan Wang

**Affiliations:** Department of Paediatrics, Pu’er People’s Hospital, Pu’er City, Yunnan Province, China

**Keywords:** Prader-Willi syndrome, Endocrine metabolism, Body fat distribution, Appetite, Memory, Correlation

## Abstract

**Objective:**

To explore the correlations between endocrine-metabolic characteristics and body fat distribution, appetite, growth, and memory in children with Prader-Willi Syndrome (PWS).

**Methods:**

Forty-six children with PWS and forty-six with simple obesity were studied, alongside a healthy control group. Researchers measured physical development, hyperphagia, memory, hormone levels, and lipid profiles. They compared endocrine-metabolic differences across groups and examined correlations between these indicators and body fat, appetite, growth, and memory in children with PWS.

**Results:**

The PWS group presented overweight, growth retardation, increased body fat, elevated hyperphagia scores and reduced memory scores relative to simple obesity and healthy control groups, with distinct plasma metabolic and endocrine profiles (higher ghrelin, Triglycerides [TG], Total Cholesterol [TC], Low-Density Lipoprotein Cholesterol [LDL-C], Homeostatic Model Assessment of Insulin Resistance [HOMA-IR] and C-peptide; lower Insulin-like Growth Factor-1 [IGF-1], Triiodothyronine [T3], Thyroxine [T4], Thyroid-Stimulating Hormone [TSH] and High-Density Lipoprotein Cholesterol [HDL-C]). Correlation analyses confirmed positive associations of ghrelin, cortisol, and HOMA-IR with Fat Mass Index (FMI); of cortisol and HOMA-IR with hyperphagia score; of TSH, TG, HOMA-IR, and C-peptide with Body Mass Index (BMI); and of IGF-1 and T3 with memory score, as well as negative associations of IGF-1 with FMI and hyperphagia score; of HDL-C with BMI; and of ghrelin with memory score (all p < 0.05).

**Conclusion:**

Children with PWS present major endocrine-metabolic abnormalities associated with abnormal body fat, hyperphagia, growth retardation, and memory impairment, which help clarify multisystem damage in hereditary obesity and guide targeted clinical interventions.

## Highlights

• Ghrelin levels in the PWS group were higher than those in the obese control group.
• The levels of insulin-like Growth Factor-1 (IGF-1), T3, T4, TSH in the PWS group were lower than in the healthy control group.
• Children with PWS exhibit distinct endocrine-metabolic characteristics closely associated with body fat distribution, appetite, growth, and memory function.

## Introduction

Prader-Willi Syndrome (PWS) is a rare genetic disorder characterized by obesity. Its pathogenesis is associated with genomic imprinting abnormalities.^1^ The most common cause is the loss of gene expression in the paternal chromosome 15q11-q13 imprinting region, with a global incidence of approximately 1/30,000 to 1/10,000.^2^ In recent years, growing evidence suggests that PWS is one of the leading causes of syndromic obesity.^3^ Clinical manifestations vary across developmental stages.^4^ Affected infants typically exhibit hypotonia, feeding difficulties, and delays in cognitive and motor development. During childhood, symptoms shift to hyperphagia, which often leads to severe obesity.^5^^,^^6^ In adolescence, hypogonadism may become apparent, and characteristic facial features are present at all stages.^7^ Patients with PWS frequently exhibit multiple endocrine-metabolic abnormalities, which significantly impact their quality of life and overall life expectancy.^8^^,^^9^

The endocrine-metabolic systems are integral to human growth, development, and metabolic processes. In pediatric cases of genetic obesity, there is a marked dysregulation of these systems, with insulin resistance emerging as a key indicator of metabolic dysfunction.^3^ The development of obesity is contingent upon an energy imbalance, characterized by a rate of triglyceride synthesis and fat storage that surpasses the rate of fat mobilization and utilization. This imbalance results in the excessive accumulation of adipose tissue and the atypical fat distribution patterns observed in PWS,^10^ indicating potential abnormalities in fat mobilization, oxidation, or triglyceride metabolism. The accumulation of excess fat diminishes insulin sensitivity in target cells,^11^ prompting compensatory hyperinsulinemia to preserve glucose homeostasis. Over time, this establishes a vicious cycle that severely disrupts glucose metabolism. Simultaneously, lipid metabolism is significantly dysregulated, evidenced by elevated levels of Triglycerides (TG) and free fatty acids, alongside reduced High-Density Lipoprotein Cholesterol (HDL-C), thereby substantially increasing the risk of cardiovascular disease.^12^^,^^13^ Research has also indicated a predominance of peripheral fat distribution in individuals with PWS,^14^ which may further influence insulin sensitivity or resistance. An early study in adult females with PWS reported a selective reduction in visceral fat, possibly contributing to decreased insulin levels via enhanced hepatic insulin clearance and altered triglyceride metabolism.^15^ Body composition studies demonstrate that, compared to Body Mass Index (BMI)-matched controls, children with PWS have lower skeletal muscle mass but comparable abdominal adipose tissue.^3^ Moreover, children with PWS exhibit lower energy expenditure,^3^^,^^16^ likely due to diminished muscle mass, hypotonia, and a resulting reduction in resting metabolic rate and physical activity, ultimately leading to decreased total energy metabolism. Overfeeding is one of the most obvious symptoms in children with PWS after the age of 2-years, and the subsequent obesity and related metabolic disorders (diabetes mellitus, dyslipidemia, obstructive sleep apnea, and cardiovascular disease) are important factors affecting the long-term survival of these children.^7^^,^^17^ Endocrine-metabolic abnormalities persist throughout the course of the disease and involve multiple hormonal systems, including growth hormone, thyroid hormones, and sex hormones, thereby directly affecting height growth, body fat distribution, and pubertal development.^18^ Importantly, memory ‒ a key component of cognitive development in children ‒ also appears to be intricately linked with genetic obesity. Chronic inflammation, insulin resistance, and oxidative stress resulting from obesity can impair neurobiological functions, potentially affecting structures such as the hippocampus that are essential for memory.^19^

Consequently, a comprehensive examination of the relationships between endocrine-metabolic function and body fat distribution, appetite, growth, and memory in children with PWS is instrumental in elucidating the underlying pathophysiological mechanisms of the condition. Such research establishes a scientific foundation for the formulation of more precise and individualized prevention and treatment strategies. This holds substantial clinical relevance for optimizing health outcomes and improving the well-being of affected individuals.

## Materials and methods

### Research subjects

A total of 46 children diagnosed with PWS who received treatment at Pu’er People’s Hospital between January 2018 and December 2023 were prospectively enrolled in this study. Inclusion Criteria: 1) Patients aged 8‒18 years; 2) Diagnosis of PWS based on the “Chinese Expert Consensus on Diagnosis and Management of Prader-Willi Syndrome (2015)” issued by the Endocrinology and Genetic Metabolism Group of the Chinese Pediatric Society. For children with typical clinical manifestations, genetic testing confirming a paternal 15q11-q13 deletion or maternal uniparental disomy establishes the diagnosis; 3) Patients diagnosed with obesity according to the “Body Mass Index Reference for Screening Overweight and Obesity in Chinese Children and Adolescents (2004)”; 4) Patients with adequately complete medical records or those eligible for telephone follow-up to verify and supplement clinical data; 5) Patients or their legal guardians having provided informed consent. Exclusion criteria: 1) Patients with negative genetic testing results or incomplete genetic workup; 2) Patients with other genetic diseases; 3) Patients with acute infectious diseases; 4) Patients who received medications affecting endocrine-metabolic functions, appetite, or growth during the study period; 5) Patients with incomplete clinical data.

Concurrently, 46-age-, gender-, and BMI-matched children diagnosed with simple obesity during the same period at Pu’er People’s Hospital were recruited as the simple obesity control group. Inclusion criteria for the simple obesity control group: 1) Patients definitively diagnosed with obesity based on the “Body Mass Index Reference for Screening Overweight and Obesity in Chinese Children and Adolescents (2004)”; 2) A confirmed diagnosis of simple obesity (primary obesity without identifiable secondary causes). This clinical diagnosis routinely excludes secondary etiologies such as hypothyroidism, Cushing’s syndrome, and monogenic obesity (e.g., leptin or leptin receptor deficiency). Except for parameters related to obesity that may be abnormal, all other indicators for the simple obesity control group were within normal ranges. Guardians of all research subjects were fully informed of the research objectives and provided written informed consent. The study protocol was approved by the Ethics Committee of Pu’er People’s Hospital.

### Demographic information

A pre-designed questionnaire was used to obtain demographic information on patients in the PWS group, children with simple obesity matched for gender, age, and BMI, and healthy controls matched for gender and age. The collected data primarily included age, gender, height, weight, BMI, appetite, growth, and memory function.

### Endocrine-metabolic assessment

All subjects underwent venous blood collection between 8:00 and 10:00 am after 6‒8 hours of fasting. Hormone levels, including ghrelin, thyroid hormones (Triiodothyronine [T3], Thyroxine [T4], and Thyroid-Stimulating Hormone [TSH]), and Cortisol (COR), were measured using chemiluminescent immunoassay. Blood glucose was assessed using the glucose oxidase method, and lipid profiles ‒ including TG, Total Cholesterol (TC), HDL-C, and Low-Density Lipoprotein Cholesterol (LDL-C) ‒ were analyzed with an automated biochemical analyzer using enzymatic methods.

### Body fat distribution

Body fat distribution was assessed using dual-energy X-Ray absorptiometry (iDXA, General Electric Lunar, 2008, Madison, USA), equipped with enCORE software version 12.6 and operated under a low radiation dose protocol (standard 3 µGy). The Fat Mass Index (FMI) was calculated by dividing total body fat mass by the square of height (kg/m^2^).

### Hyperphagia assessment

A hyperphagia questionnaire specifically designed for PWS (The Hyperphagia Questionnaire for Clinical Trials [HQ-CT]) was administered to the parents of children with PWS to assess appetite. The HQ-CT has been validated for assessing hyperphagia in individuals with PWS.^20^ This questionnaire consists of 9-items and serves as an observer-reported clinical outcome assessment tool, adapted from the hyperphagia questionnaire for PWS developed by Dykens and colleagues.^21^ The assessment was completed by parents or primary caregivers of PWS patients to evaluate the frequency and severity of hyperphagic behaviors over the preceding two weeks. It covers items such as the frequency of food-seeking behaviors, the severity of emotional outbursts when access to desired food is denied, and the persistent request for or preoccupation with food. Each item is scored from 0 to 4, yielding a total possible score of 36, with higher scores indicating more severe hyperphagia.

### Memory assessment

Memory function was evaluated using the Wechsler Memory Scale (WMS). The scale includes three components: Ⅰ. Short-term memory (picture recall, verbal paired associates, visual memory, etc.); Ⅱ. Immediate memory (digital span); Ⅲ. Long-term memory (numeric recall and accumulation). The raw scores from the three subtests were summed, and the total score was converted into a Memory Quotient (MQ) using the scale’s standardized norms. The MQ has a maximum score of 120, with higher scores indicating better memory performance. Information related to BMI was obtained on the same day as the WMS.^22^

### Statistical analysis

The collected data were analyzed using SPSS 26.0 software. Continuous variables were described as mean ± standard deviation (x̄ ± s) if normally distributed, or as median and interquartile range [M (P25, P75)] if non‑normally distributed. For comparisons between two groups, an independent‑samples *t*‑test was used when the assumptions of normality and homogeneity of variance were met; otherwise, a Welch’s *t*‑test was applied. For comparisons among multiple groups, one‑way analysis of variance was performed if assumptions of normality and homogeneity of variance were satisfied, with post‑hoc pairwise comparisons conducted using the least significant difference method. If these assumptions were violated, the Kruskal‑Wallis test was used instead. Categorical data were presented as counts and percentages (%), and group differences were assessed using the Chi‑Square (χ²) test or Fisher’s exact test when expected frequencies were below 5. Correlation analyses were performed to examine the relationships between endocrine‑metabolic indicators and measures of body fat distribution, hyperphagia, BMI, and memory scores (WMS scores). Pearson correlation coefficient was used for variables with a linear relationship when assumptions were met; otherwise, Spearman’s rank correlation coefficient was applied. p‑values were adjusted using the Bonferroni correction, with p < 0.05 considered statistically significant.

## Results

### Analysis of general clinical data

A comparative analysis was conducted among 46 children with PWS, 46 children with simple obesity, and 46 healthy children. Significant differences were observed across multiple parameters between the groups. No significant differences were found in age or gender (p > 0.05), indicating comparable demographic characteristics. The weight of children in the PWS group was significantly lower than that in the simple obesity control group but higher than in the healthy control group (p < 0.001). Their height was significantly lower than that of the simple obesity control group (p < 0.001), while their BMI was significantly higher than that of the healthy controls (p < 0.001), suggesting that children with PWS present with excess weight and growth retardation. Regarding body fat distribution, the PWS group showed higher FMI and Visceral Fat Area (VFA) compared with both control groups (FMI: p = 0.004 vs. obese controls, p < 0.001 vs. healthy controls; VFA: p = 0.357 vs. obese controls, p < 0.001 vs. healthy controls). Although the difference in VFA between the PWS group and the simple obesity control group was not statistically significant, the PWS group had significantly higher VFA than the healthy control group, indicating more severe fat accumulation in children with PWS. In terms of appetite, hyperphagia scores in the PWS group were significantly higher than those in both control groups (p < 0.001). For memory function, WMS scores were significantly lower in the PWS group compared with both control groups (p = 0.001 and p < 0.001, respectively), reflecting impaired memory function in children with PWS. Additionally, among these 46 children with PWS, 34 (73.91%) had received growth hormone therapy (data not shown in Table 1). Details are presented in Table 1.

**Table 1:** Clinical characteristics of patients with PWS and the control group.

Indicator	PWS Group (n = 46)	Obesity Control Group (n = 46)	Healthy Control Group (n = 46)	PWS vs. OB		PWS vs. Health	
				*t*/z/χ²	p-value	*t*/z/χ²	p-value
Age (years)	9.86 ± 1.33	10.43 ± 1.47	10.25 ± 1.39	1.95	0.127	1.375	0.377
Gender				0.418	0.668	1.614	0.290
Male	30 (65.22%)	27 (58.70%)	24 (52.17%)				
Female	16 (34.78%)	19 (41.30%)	22 (47.83%)				
Weight (kg)	64.36 ± 8.67	74.32 ± 10.23	38.42 ± 7.16	5.038	<0.001	15.65	<0.001
Height (m)	1.34 ± 0.15	1.57 ± 0.21	1.43 ± 0.19	6.045	<0.001	2.552	0.0134
BMI (kg/m^2^)	34.23 ± 4.71	31.56 ± 4.12	18.47 ± 2.11	2.894	0.005	20.71	<0.001
Fat Mass Index (kg/m^2^)	8.66 ± 1.50	7.83 ± 1.34	3.46 ± 0.58	2.799	0.006	21.93	<0.001
Visceral Fat Area (cm^2^)	122.31 ± 8.37	124.76 ± 9.35	87.84 ± 7.84	1.324	0.200	20.39	<0.001
Hyperphagia Score	9 (8, 10)	7 (6, 8)	4 (3, 4)	3.803	0.001	47.50	<0.001
Memory WISC-IV Score	78 (75, 82)	89 (87, 91)	91 (88, 95)	10.43	<0.001	114	<0.001

### Endocrine-metabolic characteristics of children with PWS

The concentration of ghrelin in the PWS group was 1.04 (0.94, 1.21) ng/mL, which was higher than that in the simple obesity control group (0.98 [0.89, 1.09] ng/mL, p = 0.034) but significantly lower than that in the healthy control group (1.26 [1.11, 1.40] ng/mL, p < 0.001). The level of Insulin-like Growth Factor-1 (IGF-1) in the PWS group was 132.93 (127.02, 140.99) ng/mL, which was significantly lower than the levels in the simple obesity control group (184.56 [177.51, 189.28] ng/mL) and the healthy control group (214.76 [205.09, 224.85] ng/mL) (p < 0.001 for both). For thyroid hormones, T3 and T4 levels in the PWS group were significantly lower than those in both control groups (all p < 0.001). TSH levels in the PWS group did not differ significantly from the simple obesity control group (p = 0.424) but were lower than those in the healthy control group (p = 0.003). Regarding Cortisol (COR), no significant difference was observed between the PWS group and the simple obesity control group (p = 0.719), while COR was higher in the PWS group compared with the healthy control group (p = 0.059). In terms of lipid profiles, Triglyceride (TG) levels in the PWS group were 1.43 ± 0.55 mmoL/L, which was higher than those in the simple obesity control group (1.07 ± 0.45 mmoL/L, p = 0.003) and significantly higher than those in the healthy control group (0.63 ± 0.31 mmoL/L, p < 0.001). TC and LDL-C levels followed trends similar to TG (all p < 0.05), while HDL-C levels were lower than those in the healthy controls (p = 0.009). In the PWS cohort, the Homeostatic Model Assessment of Insulin Resistance (HOMA-IR) was 3.94 (2.78, 5.93), which was significantly elevated compared to the healthy control group value of 0.98 (0.60, 1.15) (p < 0.001). Additionally, the C-peptide level in the PWS group was 3.83 ± 1.55 ng/mL, significantly higher than the levels in both the simple obesity control group and the healthy control group (both p < 0.001) (Table 2).

**Table 2:** Comparison of endocrine hormone levels among the three groups.

Indicator	PWS Group (n = 46)	Obese Control Group (n = 46)	Healthy Control Group (n = 46)	p-value (PWS vs. OB)	p-value (PWS vs. Health)
Ghrelin (ng/mL)	1.04 (0.94, 1.21)	0.98 (0.89, 1.09)	1.26 (1.11, 1.40)	0.034	<0.001
IGF-1 (ng/mL)	132.93 (127.02, 140.99)	184.56 (177.51, 189.28)	214.76 (205.09, 224.85)	<0.001	<0.001
Triiodothyronine (T3, nmoL/L)	2.57 ± 0.11	2.96 ± 0.17	2.82 ± 0.14	<0.001	<0.001
Thyroxine (T4, nmoL/L)	100.47 ± 6.34	108.34 ± 7.45	114.56 ± 8.47	<0.001	<0.001
Thyroid-Stimulating Hormone (TSH, μIU/mL)	3.76 ± 0.32	3.85 ± 0.38	4.02 ± 0.44	0.424	0.003
Cortisol (COR, nmoL/L)	364.76 ± 54.14	357.84 ± 43.76	342.78 ± 47.56	0.719	0.059
Triglycerides (TG, mmoL/L)	1.43 ± 0.55	1.07 ± 0.45	0.63 ± 0.31	0.003	<0.001
Total Cholesterol (TC, mmoL/L)	4.96 ± 1.05	4.27 ± 0.74	4.23 ± 0.58	0.002	<0.001
High-Density Lipoprotein Cholesterol (HDL-C, mmoL/L)	1.24 ± 0.25	1.13 ± 0.23	1.38 ± 0.22	0.047	0.009
Low-Density Lipoprotein Cholesterol (LDL-C, mmoL/L)	3.16 ± 1.07	2.64 ± 0.51	2.34 ± 0.45	0.002	<0.001
Insulin Resistance Index (HOMA-IR)	3.94 (2.78, 5.93)	3.86 (2.86, 4.79)	0.98 (0.60, 1.15)	0.706	<0.001
C-peptide	3.83 ± 1.55	1.65 ± 0.58	1.13 ± 0.41	< 0.001	<0.001

## Correlation analysis of endocrine-metabolic factors with body fat distribution, appetite, growth, and memory in children with PWS

**Fig. 1:**
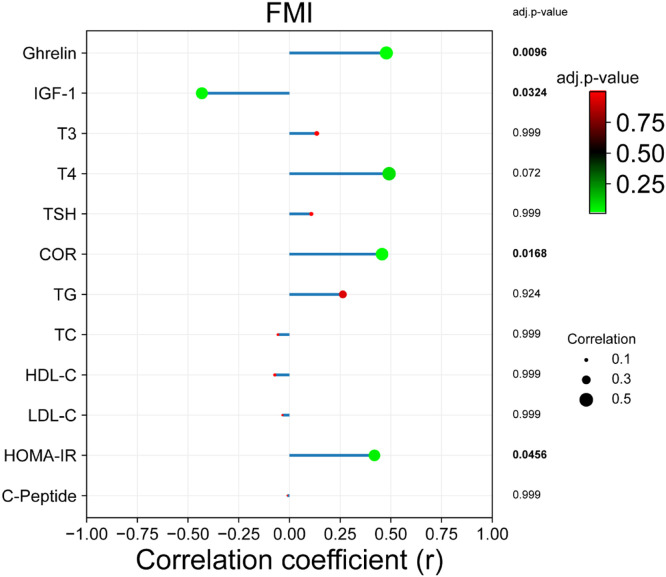
Correlation Analysis of Endocrine-Metabolism and FMI in Children with PWS (n = 46). Correlations are displayed using a lollipop plot, where point position indicates the direction (left: negative, right: positive), point size corresponds to the absolute correlation coefficient, and color represents the p-value. Spearman correlation was used for ghrelin, IGF-1, and HOMA-IR; Pearson correlation was applied for all other indicators.

In children with hereditary obesity, endocrine-metabolic indicators showed significant correlations with body fat distribution. Ghrelin was positively correlated with FMI (*r*ₛ = 0.477, adj.p = 0.0096), suggesting that higher ghrelin levels were accompanied by increased body fat. IGF-1 was negatively correlated with FMI (*r*ₛ = -0.4319, adj.p = 0.0324), indicating that higher IGF-1 levels were associated with lower body fat. COR was positively correlated with FMI (*r* = 0.4565, adj.p = 0.0168), demonstrating an association between the two. Furthermore, the HOMA-IR showed a significant positive correlation with FMI (*r* = 0.4189, adj.p = 0.0456), suggesting that the degree of insulin resistance co-occurred with increased body fat. As shown in Fig. 1, T3, T4, TSH, TG, TC, HDL-C, LDL-C, and C-peptide showed weak correlations with FMI.

**Fig. 2:**
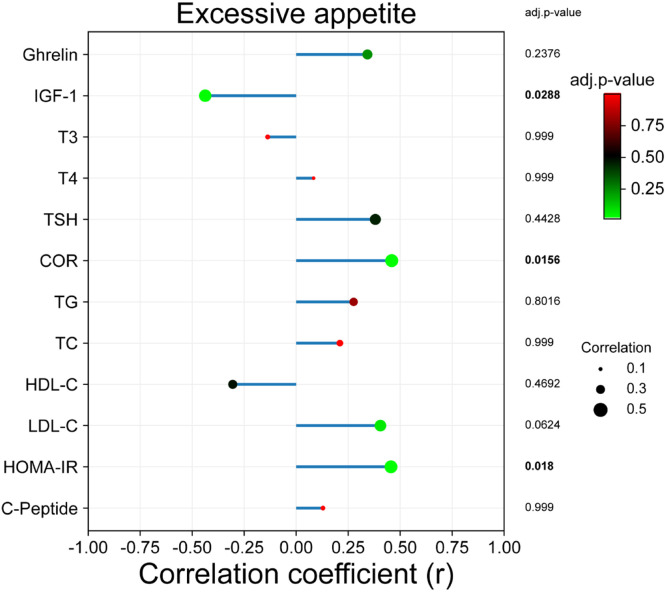
Correlation Analysis of Endocrine-Metabolism and Hyperphagia in Children with PWS (n = 46). Correlations are displayed using a lollipop plot, where point position indicates the direction (left: negative, right: positive), point size corresponds to the absolute correlation coefficient, and color represents the p-value. Spearman correlation was used for ghrelin, IGF-1, and HOMA-IR; Pearson correlation was applied for all other indicators.

The correlations between endocrine-metabolic indicators and hyperphagia were further analyzed (Fig. 2). IGF-1 was negatively correlated with hyperphagia score (*r* = -0.4376, adj.p = 0.0288), indicating that children with PWS and higher hyperphagia scores had lower IGF-1 levels. COR was positively correlated with hyperphagia score (*r* = 0.4592, adj.p = 0.0156), with higher COR levels observed in children with more severe hyperphagia. Additionally, HOMA-IR was positively correlated with hyperphagia score (*r* = 0.4556, adj.p = 0.0180), indicating that a higher degree of insulin resistance may be associated with more pronounced hyperphagia. Weak correlations were found for T3, T4, TSH, TG, TC, HDL-C, LDL-C, and C-peptide with hyperphagia score.

**Fig. 3:**
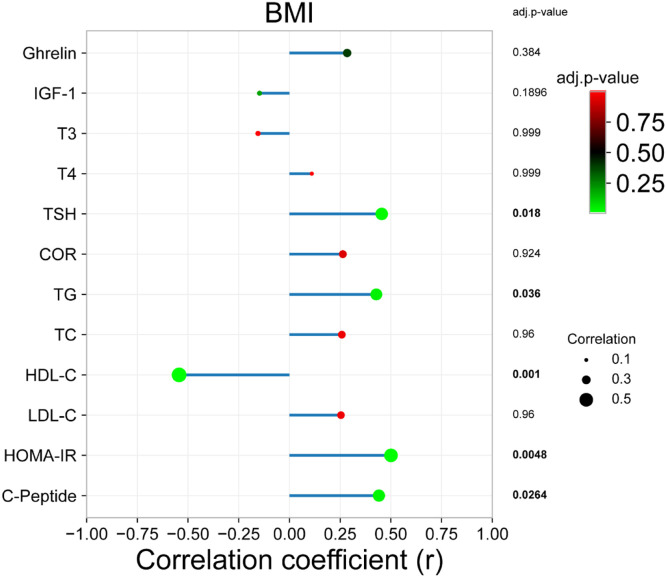
Correlation Analysis of Endocrine-Metabolism and BMI in Children with PWS (n = 46). Correlations are displayed using a lollipop plot, where point position indicates the direction (left: negative, right: positive), point size corresponds to the absolute correlation coefficient, and color represents the p-value. Spearman correlation was used for ghrelin, IGF-1, and HOMA-IR; Pearson correlation was applied for all other indicators.

Furthermore, in children with hereditary obesity, endocrine-metabolic indicators were significantly correlated with growth. TSH was positively correlated with BMI (*r* = 0.4547, adj.p = 0.0180), suggesting that TSH levels may increase with higher BMI. TG was also positively correlated with BMI (*r* = 0.4280, adj.p = 0.0360), indicating an association between elevated TG levels and increased BMI. HDL-C was negatively correlated with BMI (*r* = -0.5441, adj.p = 0.0012), suggesting that children with higher HDL-C levels typically had lower BMI. Moreover, both HOMA-IR and C-peptide were positively correlated with BMI (*r* = 0.5004 and *r* = 0.4410, respectively; both adj.p < 0.05), indicating that the degree of insulin resistance and C-peptide level co-occurred with increased BMI. These findings reveal close associations between endocrine-metabolic indicators and growth-related parameters (Fig. 3).

**Fig. 4:**
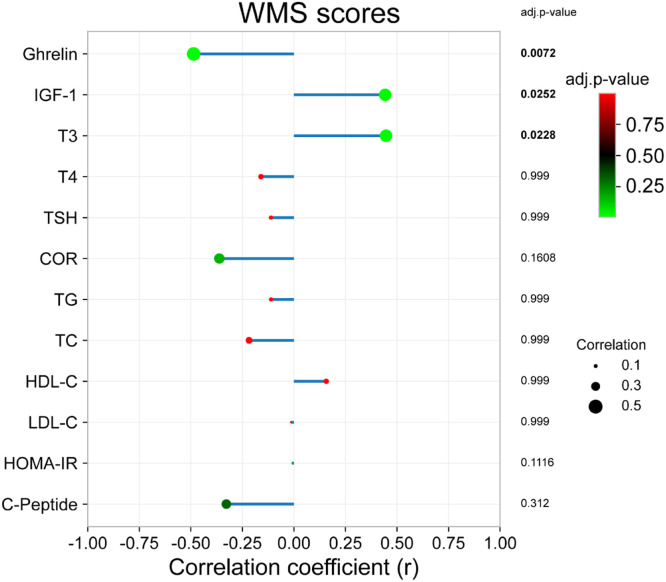
Correlation Analysis of Endocrine-Metabolism and Memory in Children with PWS (n = 46). Correlations are displayed using a lollipop plot, where point position indicates the direction (left: negative, right: positive), point size corresponds to the absolute correlation coefficient, and color represents the p-value. Spearman correlation was used for ghrelin, IGF-1, and HOMA-IR; Pearson correlation was applied for all other indicators.

Finally, ghrelin was negatively correlated with memory WMS score (*r* = -0.4867, adj.p = 0.0072), suggesting that individuals with higher ghrelin levels may have lower memory scores. IGF-1 was positively correlated with memory WMS score (*r* = 0.4424, adj.p = 0.0252), indicating that higher IGF-1 levels may be associated with better memory performance. T3 showed a significant positive correlation with memory WMS score (*r* = 0.4466, adj.p = 0.0228), demonstrating a positive link between thyroid hormone levels and memory score. Correlations between other indicators and memory were weak and not statistically significant (Fig. 4).

## Discussion

PWS is a multifaceted genetic disorder impacting the endocrine and nervous systems, as well as metabolism and behavior.^9^ The syndrome is often marked by endocrine irregularities, such as hypogonadism, dysfunction of the growth hormone/IGF-1 axis, hypothyroidism, and central adrenal insufficiency. PWS commonly manifests with early-onset hyperphagia and food-seeking behaviors, which progressively result in severe obesity.^23^ In the early stage, PWS is characterized by hypotonia and growth retardation, subsequently leading to obesity and hyperphagia.^24^ Patients with PWS exhibit hypothalamic dysfunction, which may contribute to a range of endocrine disorders, including growth hormone deficiency, hypogonadism, hypothyroidism, central adrenal insufficiency, and reduced Bone Mineral Density (BMD). Beyond hypothalamic dysfunction and impaired satiety, individuals with PWS often experience decreased resting energy expenditure, increasing the risk of obesity, metabolic syndrome, and type 2 diabetes mellitus.^7^ Accordingly, the present investigation was designed to analyze and contrast clinical parameters among three pediatric groups: children with PWS, children with simple obesity, and healthy controls, with the aim of elucidating the endocrine-metabolic profile in PWS and examining its correlations with body fat distribution, appetite, growth, and memory function.

Endocrine-metabolic dysregulation is a central driver of abnormal adiposity in hereditary obesity. The PWS group demonstrated significantly higher FMI and VFA than the healthy control group, and a higher FMI than the simple obesity control group, indicating more severe overall and visceral fat accumulation in children with PWS. Metabolically, children with PWS exhibited elevated TG, TC, and LDL-C levels compared to both control groups, alongside lower HDL-C levels than healthy controls. Dyslipidemia is associated with imbalances in lipid metabolism and transport, as well as excessive subcutaneous and visceral fat deposition. Elevated TG and LDL-C may reflect increased hepatic steatosis and contribute to aberrant body fat distribution.^12^^,^^25^ While Cummings et al. reported elevated plasma ghrelin in PWS,^26^ the present study observed an opposite trend, with PWS levels being lower than in healthy controls. This discrepancy may be attributed to differences in sample characteristics, potential comorbidity effects, or recent lifestyle factors. This finding highlights the need for larger, multicenter studies to clarify the role of ghrelin in PWS across diverse populations and developmental stages. Nevertheless, a positive correlation was found between ghrelin and FMI in this cohort, suggesting a potential link between this appetite-regulating hormone and fat accumulation, possibly mediated through its effects on hunger and food intake. HOMA-IR and C-peptide levels were significantly elevated in the PWS group, confirming an insulin-resistant phenotype. Insulin resistance impairs adipocyte function, disrupting lipid storage and lipolysis,^11^ which may promote fat storage and adipose tissue inflammation, thereby altering body fat distribution. Furthermore, IGF-1 was negatively correlated with FMI. The significantly lower IGF-1 levels in children with PWS may indirectly influence lipolysis through fat metabolism pathways, contributing to adiposity. An important consideration is that growth hormone therapy, a common intervention in PWS, profoundly affects IGF-1 levels and body composition. The present study did not account for GH treatment status (e.g., prevalence, duration, dose), which represents a potential confounding factor in analyzing IGF-1 associations. Additionally, comorbidities like sleep apnea and reduced physical activity in PWS may concurrently influence lipid metabolism and fat distribution, necessitating their control in future studies to clarify the specific roles of IGF-1 and GH therapy.

Dykens et al. introduced an innovative approach for assessing hyperphagia in individuals with PWS. Consistent with their findings, the present study observed that children with PWS had markedly elevated levels of hyperphagia, with scores significantly surpassing those of the other two comparative groups. After adjustment for multiple endocrine-metabolic indicators, the correlation between ghrelin and hyperphagia score was not statistically significant. However, ghrelin dysregulation may still relate to hypothalamic appetite center dysfunction, indirectly influencing food intake and obesity severity.^27^ A negative correlation was observed between IGF-1 and hyperphagia score, suggesting that low IGF-1 levels may be linked to appetite dysregulation via central pathways, though not as a direct modulator.^28^ Other hormones in the endocrine system, such as thyroid hormones and COR, also indirectly participate in appetite regulation.^29^^,^^30^ Abnormal activity in the brain's reward system may further exacerbate strong food cravings and related behavioral problems in PWS patients in response to food-related stimuli.^31^ Critically, the underlying genetic defect in PWS (e.g., 15q11-q13 deletion) may simultaneously disrupt hormonal axes and neural reward circuits, while environmental factors like food accessibility also modulate appetite. Thus, the relationship between hormones and hyperphagia is likely multifactorial and confounded, precluding simple causal interpretation.

Growth and development are modulated by multiple factors, with endocrine-metabolic indicators playing a significant role. Children with PWS in this study were significantly shorter than the simple obesity controls, exhibiting a profile of excess weight with growth retardation. TSH showed a positive correlation with BMI, suggesting thyroid function may be involved in growth regulation in PWS. Abnormal thyroid hormone levels and disrupted energy metabolism can impair the secretion and action of growth hormone and other anabolic hormones, hindering linear growth. Positive correlations of TG, HOMA-IR, and C-peptide with BMI indicate that metabolic disturbances promoting fat accumulation also interfere with normal growth processes. Van et al. reported lower IGF-1 levels in PWS patients (median BMI 27.5 kg/m^2^) without a significant correlation with BMI, implying that IGF-1 reduction is not solely obesity-dependent.^32^ Similarly, no correlation between IGF-1 and BMI was found in the present PWS cohort. It must be considered that the genetic basis of PWS may concurrently cause endocrine abnormalities and growth impairment, while factors like nutritional intake and physical activity also contribute. Therefore, associations between endocrine markers and growth parameters should be interpreted within a multifactorial framework, acknowledging potential interactions.

Regarding memory function, children with PWS had significantly lower WMS scores than both control groups, indicating notable memory impairment. Endocrine-metabolic indicators showed several associations with memory performance. Ghrelin was negatively correlated with memory scores; elevated levels may impair memory by affecting neurotransmitter dynamics or neuronal plasticity. IGF-1 was positively correlated with memory, suggesting that lower levels might compromise processes like neurogenesis, synaptic formation, and neuronal survival. Thyroid hormones (T3 and T4) were positively correlated with memory, indicating that their deficiency in PWS could disrupt neuronal energy metabolism and signaling, adversely affecting memory. The interplay between thyroid hormones and insulin signaling may also be important for cerebral glucose metabolism and memory function.^33^ Elevated COR levels have been negatively correlated with memory performance in PWS.^34^^,^^35^ As the hippocampus is crucial for memory and is sensitive to glucocorticoids, high COR may induce oxidative stress and inflammation, damaging hippocampal structure and function.^36^^,^^37^

The findings identify key endocrine-metabolic abnormalities in hereditary obesity (e.g., reduced IGF-1, altered ghrelin, thyroid hormone deficiency, insulin resistance, dyslipidemia), which may serve as potential biomarkers for clinical screening and monitoring. Correlations between these indicators and body fat distribution, appetite, growth, and memory suggest avenues for targeted intervention ‒ for instance, addressing IGF-1 deficiency to support growth, modulating insulin resistance and lipids to mitigate adiposity, and targeting appetite-regulating pathways to control hyperphagia. The multi-system involvement in PWS underscores the need for integrated management strategies that address weight, endocrine function, growth, and cognition concurrently.

This study has several limitations. First, its single-center design with participants from one region may introduce selection bias and limit generalizability; multi-center studies with larger cohorts are needed. Second, the modest sample size (46-per-group) may limit statistical power, particularly for detecting weaker associations. Third, the cross-sectional nature precludes causal inferences; longitudinal or interventional studies are required to elucidate mechanisms. Fourth, interactions among endocrine-metabolic factors were not explored in depth; the complex pathophysiology of PWS likely involves synergistic effects across multiple systems, warranting further network-based analyses. Fifth, key treatment-related information, such as the mean duration of growth hormone therapy, was not collected or analyzed in this study. This represents an important limitation, as it precludes a comprehensive assessment of the impact of treatment-related factors on the study outcomes. Future studies should incorporate the collection and stratified analysis of treatment-related data.

In conclusion, this study demonstrates that children with hereditary obesity (PWS) exhibit significant endocrine-metabolic disturbances, which are closely correlated with abnormal body fat distribution, hyperphagia, growth retardation, and memory impairment. These findings provide insight into the pathophysiology of hereditary obesity and highlight potential biomarkers and intervention targets. Future multi-center, prospective studies with larger samples are necessary to validate these correlations, establish causal relationships, and inform comprehensive, precision-based strategies for the management of hereditary obesity.

## Data available

Data are available from the corresponding author on request.

## Ethics statement

The present study was approved by the Ethics Committee of Pu’er People’s Hospital (n°201708PE26), and written informed consent was provided by all patients prior to the study start. All procedures were performed in accordance with the ethical standards of the Institutional Review Board and The Declaration of Helsinki, and its later amendments or comparable ethical standards.

## Authors’ contributions

Conceptualization, Ying Zhang and Xiang Li; data curation, Jian Wen and Yong Rao; formal analysis, Xiang Li and Juan Wang; investigation, Ying Zhang; methodology, Jian Wen and Yong Rao; writing-original draft preparation, Ying Zhang and Xiang Li; writing-review and editing, Juan Wang. All authors have read and agreed to the published version of the manuscript.

## Funding

Not applicable.

## Acknowledgments

Not applicable.

## Declaration of competing interest

The authors declare no conflicts of interest.

## References

[1] Shepherd D.A., Vos N., Reid S.M. (2020). Growth trajectories in genetic subtypes of Prader-Willi Syndrome. Genes (Basel).

[2] Tsai J.H., Scheimann A.O., McCandless S.E., Strong T.V., Bridges J.F.P. (2018). Caregiver priorities for endpoints to evaluate treatments for Prader-Willi syndrome: a best-worst scaling. J Med Econ.

[3] Irizarry K.A., Miller M., Freemark M., Haqq A.M. (2016). Prader Willi Syndrome: genetics, metabolomics, hormonal function, and new approaches to therapy. Adv Pediatr.

[4] Butler M.G., Lee J., Cox D.M. (2016). Growth charts for Prader-Willi syndrome during Growth hormone treatment. Clin Pediatr (Phila).

[5] Butler M.G., Kimonis V., Dykens E. (2018). Prader-Willi syndrome and early-onset morbid obesity NIH rare disease consortium: a review of natural history study. Am J Med Genet A.

[6] Singh P., Mahmoud R., Gold J.A. (2018). Multicentre study of maternal and neonatal outcomes in individuals with Prader-Willi syndrome. J Med Genet.

[7] Angulo M.A., Butler M.G., Cataletto M.E. (2015). Prader-Willi syndrome: a review of clinical, genetic, and endocrine findings. J Endocrinol Invest.

[8] Tauber M., Hoybye C. (2021). Endocrine disorders in Prader-Willi syndrome: a model to understand and treat hypothalamic dysfunction. Lancet Diabetes Endocrinol.

[9] Heksch R., Kamboj M., Anglin K., Obrynba K. (2017). Review of Prader-Willi syndrome: the endocrine approach. Transl Pediatr.

[10] Meaney F.J., Butler M.G. (1989). Characterization of obesity in the Prader-Labhart-Willi Syndrome: fatness patterning. Med Anthropol Q.

[11] Talebizadeh Z., Butler M.G. (2005). Insulin resistance and obesity-related factors in Prader-Willi syndrome: comparison with obese subjects. Clin Genet.

[12] Tanaka Y., Abe Y., Oto Y. (2013). Characterization of fat distribution in Prader-Willi syndrome: relationships with adipocytokines and influence of growth hormone treatment. Am J Med Genet A.

[13] Coupaye M., Lorenzini F., Lloret-Linares C. (2013). Growth hormone therapy for children and adolescents with Prader-Willi syndrome is associated with improved body composition and metabolic status in adulthood. J Clin Endocrinol Metab.

[14] Brambilla P., Bosio L., Manzoni P., Pietrobelli A., Beccaria L., Chiumello G. (1997). Peculiar body composition in patients with Prader-Labhart-Willi syndrome. Am J Clin Nutr.

[15] Goldstone A.P., Thomas E.L., Brynes A.E. (2001). Visceral adipose tissue and metabolic complications of obesity are reduced in Prader-Willi syndrome female adults: evidence for novel influences on body fat distribution. J Clin Endocrinol Metab.

[16] Rubin D.A., Nowak J., McLaren E., Patino M., Castner D.M., Dumont-Driscoll M.C. (2015). Nutritional intakes in children with Prader-Willi syndrome and non-congenital obesity. Food Nutr Res.

[17] Muscogiuri G., Barrea L., Faggiano F. (2021). Obesity in Prader-Willi syndrome: physiopathological mechanisms, nutritional and pharmacological approaches. J Endocrinol Invest.

[18] Other views of the future pediatrician. J Pediatr. 1986;109(1):225-9. 10.1016/s0022-3476(86)80607-2 3723236

[19] Unamuno X., Gomez-Ambrosi J., Rodriguez A., Becerril S., Fruhbeck G., Catalan V. (2018). Adipokine dysregulation and adipose tissue inflammation in human obesity. Eur J Clin Invest.

[20] Matesevac L., Vrana-Diaz C.J., Bohonowych J.E., Schwartz L., Strong T.V. (2023). Analysis of hyperphagia questionnaire for Clinical trials (HQ-CT) scores in typically developing individuals and those with Prader-Willi syndrome. Sci Rep.

[21] Goldstone A.P. (2007). Prader-Willi syndrome: obesity due to genomic imprinting. Curr Genomics.

[22] Li X., Wang T., Tao Y., Wang X., Zhang J. (2021). The role of ferroptosis in diabetic kidney disease: mechanisms and therapeutic potential. Cell Death Dis.

[23] Crino A., Fintini D., Bocchini S., Grugni G. (2018). Obesity management in Prader-Willi syndrome: current perspectives. Diabetes Metab Syndr Obes.

[24] Miller J.L. (2012). Approach to the child with prader-willi syndrome. J Clin Endocrinol Metab.

[25] Ebbert J.O., Jensen M.D. (2013). Fat depots, free fatty acids, and dyslipidemia. Nutrients.

[26] Cummings D.E., Clement K., Purnell J.Q. (2002). Elevated plasma ghrelin levels in Prader Willi syndrome. Nat Med.

[27] Purtell L., Sze L., Loughnan G. (2011). In adults with Prader-Willi syndrome, elevated ghrelin levels are more consistent with hyperphagia than high PYY and GLP-1 levels. Neuropeptides.

[28] Damen L., Elizabeth M.S.M., Donze S.H., van den Berg S.A.A., de Graaff L.C.G., ACS H-K (2022). Free insulin-like growth factor (IGF)-I in children with PWS. J Clin Med.

[29] Sawicka-Gutaj N., Zawalna N., Gut P., Ruchala M. (2022). Relationship between thyroid hormones and central nervous system metabolism in physiological and pathological conditions. Pharmacol Rep.

[30] Straub R.H. (2014). Interaction of the endocrine system with inflammation: a function of energy and volume regulation. Arthritis Res Ther.

[31] Strelnikov K., Debladis J., Salles J. (2023). Amygdala hyperactivation relates to eating behaviour: a potential indicator of food addiction in Prader-Willi syndrome. Brain Commun.

[32] van Nieuwpoort I.C., Twisk J.W.R., Curfs L.M.G., Lips P., Drent M.L. (2018). Body composition, adipokines, bone mineral density and bone remodeling markers in relation to IGF-1 levels in adults with Prader-Willi syndrome. Int J Pediatr Endocrinol.

[33] Jahagirdar V., McNay E.C. (2012). Thyroid hormone's role in regulating brain glucose metabolism and potentially modulating hippocampal cognitive processes. Metab Brain Dis.

[34] Oto Y., Matsubara K., Ayabe T. (2018). Delayed peak response of cortisol to insulin tolerance test in patients with Prader-Willi syndrome. Am J Med Genet A.

[35] Damen L., Donze S.H., Grootjen L.N., Hokken-Koelega A.C.S. (2021). Long-term cortisol levels in hair of children and adolescents with Prader-Willi Syndrome. Psychoneuroendocrinology.

[36] Dronse J., Ohndorf A., Richter N. (2023). Serum cortisol is negatively related to hippocampal volume, brain structure, and memory performance in healthy aging and Alzheimer's disease. Front Aging Neurosci.

[37] Almela M., Hidalgo V., van der Meij L., Pulopulos M.M., Villada C., Salvador A. (2014). A low cortisol response to acute stress is related to worse basal memory performance in older people. Front Aging Neurosci.

